# Outcome and experience of arthroscopic lateral retinacular release combined with lateral patelloplasty in the management of excessive lateral pressure syndrome

**DOI:** 10.1186/s13018-021-02229-4

**Published:** 2021-01-22

**Authors:** Cheng-Liang Wang, Ji-Bin Chen, Te Li

**Affiliations:** 1 Third Ward of Orthopedic Department, CR & WISCO General Hospital, Wuhan University of Science and Technology, Wuhan, 430080 China; 2 Department of Orthopedic Surgery, Wuhan Hanyang Hospital, Wuhan University of Science and Technology, No.53 Moshuihu Road, Hanyang District, Wuhan, Hubei Province China; 3 Department of Orthopedic Surgery, General Hospital of Central Theater Command, No.68 Huangpu Road, Jiangan District, Wuhan, Hubei Province China

**Keywords:** Arthroscopic surgery, Excessive lateral pressure syndrome, Lateral patella retinaculum, Release, Arthroplasty

## Abstract

**Background:**

Only a few studies have described the effect of full arthroscopic surgery in treatment of excessive lateral pressure syndrome (ELPS). Therefore, the purpose of this study was to assess the clinical efficacy and experience of total arthroscopic lateral retinacular (LR) release and lateral patelloplasty for the treatment of ELPS.

**Methods:**

A total of 73 patients (88 knees) with ELPS underwent arthroscopic LR release and lateral patelloplasty. The visual analogue scale (VAS), Kujala score, Lysholm scores, patella medial pushing distance, patellar tilt angle (PTA), and lateral patellofemoral angle (LPFA) were measured and evaluated before and after surgery.

**Results:**

Follow-up ranged from 12 to 36 months with an average of 24 ± 5.8 months. The VAS was significantly lower at the last follow-up than before surgery (*P* < 0.01). The patella medial pushing distance, Kujala score, Lysholm score, PTA, and LPFA were significantly higher at the last follow-up than before surgery (*P* < 0.01, respectively). Years and lateral patella Outerbridge classification at the last follow-up have negative correlation with Kujala score, Lysholm score, Patella medial pushing distance, PTA, and LPFA (*P* < 0.01, respectively) and have positive correlation with VAS (*P* < 0.01, respectively). Related complications were not reported.

**Conclusions:**

Full arthroscopic LR release combined with lateral patelloplasty in the treatment of ELPS is an effective minimally invasive method, which can effectively correct anomalies of force line and skeleton of patella, relieve pain, and restore knee joint motor function with less complications.

## Background

Excessive lateral pressure syndrome (ELPS), also known as lateral patellar compression syndrome, is featured by long-term lateral tilt of the patella without subluxation or dislocation and adaptive shortening of lateral retinaculum (LR), which results in long-term stress imbalance of the medial and lateral patellofemoral articular surface, increased lateral patellofemoral joint pressure, and ultimately causes a series of pain syndromes [1]. The lateral tilt of the patella is secondary to the contracture of the LR, whose pathogenesis is related to the increased lateral patellofemoral joint stress [2]. ELPS is one of the common causes of anterior knee pain, which can cause severe patellofemoral joint dysfunction [3].

LR release for most patients with patellar tilt and mild joint lesions can achieve certain efficacy [4]. The LR release can restore abnormally inclined patella and protect lateral patellofemoral articular surface from damaging [5]. The LR release includes different procedures such as open release, percutaneous release, arthroscopic-assisted release, and total arthroscopic release. With the development of arthroscopic techniques, total arthroscopic release has been increasingly accepted by most surgeons [6]. Arthroscopic LR release has become the main method in treatment of ELPS [5].

Lateral patelloplasty repairs lateral patellar morphology to correct abnormal motion trajectory of patellofemoral joint. Early open lateral patelloplasty was used for lateral patellar compression syndrome treatment [7]. With the development of arthroscopic technology, arthroscopic lateral patelloplasty was used in the treatment of lateral patellofemoral osteoarthritis and lateral patellar impingement syndrome [8, 9].

The late stage of ELPS is accompanied by different degrees of patellofemoral arthritis and degenerative changes in the shape of the lateral patella. At present, there are few reports on full arthroscopic surgery in treatment of ELPS [10]. Therefore, the purpose of this study was to assess the clinical efficacy and experience of total arthroscopic LR release combined with lateral patelloplasty for the treatment of ELPS. It was assumed that LR release combined with lateral patelloplasty could not only correct abnormal force line, but also improve abnormal morphology of the lateral patella, correct mechanical abnormality and eventually relieve anterior knee pain and improve patellofemoral joint function. So the clinical outcomes such as patella medial mobility test, Kujala score, visual analogue scale (VAS), patellar tilt angle (PTA), lateral patellofemoral angle (LPFA), and Lysholm scores would be ameliorated after surgery.

## Materials and methods

### Patients

**Fig. 1:**
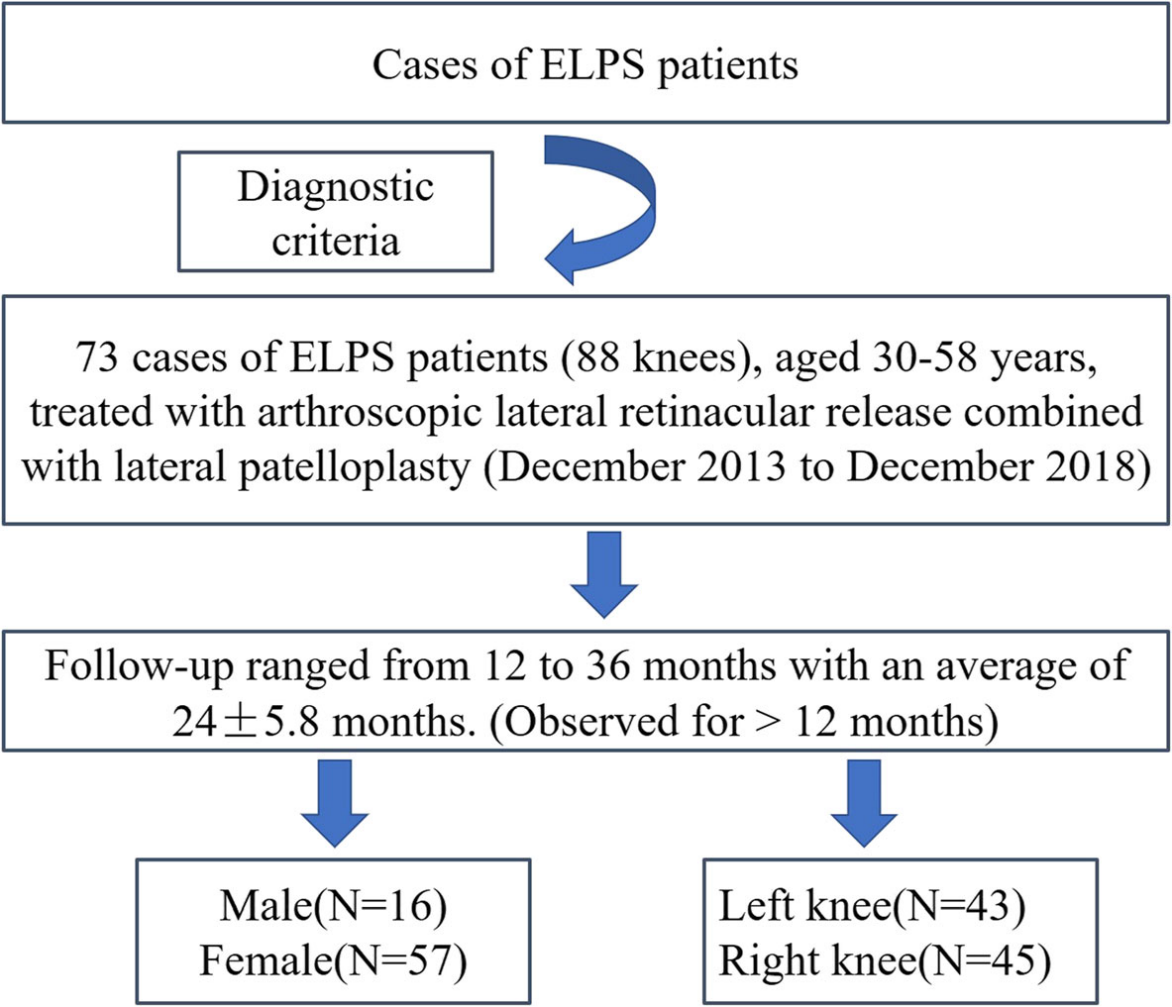
Cases of ELPS and follow-up period

This study is a retrospective study. A total of 73 patients (88 knees) of ELPS patients in the authors’ hospitals from December 2013 to December 2018 who had intractable pain of anterior knee at least for 6 months were enrolled in this study, including 16 males and 57 females, aged 30–58 years, with 43 knees in the left knee and 45 knees in the right knee (Fig. 1). The diagnostic criteria were as follows (Table 1). All patients underwent conservative treatment at least 6 months, such as quadriceps muscle strength training, medial patella pushing exercise, non-steroidal anti-inflammatory drugs, and other rehabilitation exercises, local block, etc., and the symptoms were not ameliorated.

**Table 1:** The diagnostic criteria of ELPS

Diagnostic criteria	
① All patients reported persistent pain and crepitus of anterior knee when going up and down stairs and squatting. ② The patellar medial shift [11] was less than 5 mm. ③ The anterior knee pain was induced by 30 degrees of knee flexion. ④ Patellar peripheral finger tenderness was touched on the surface of LR. ⑤ X-ray patella axial images showed obviously tilted outward patella, which was characterized by Wiberg type III or IV patellar [12]. ⑥ The knee flexion 20 degree CT examination showed abnormalities of PTA and LPFA [13, 14].	

### Ethical statement

The present study complied with the World Medical Association Declaration of Helsinki-Ethical Principles for Medical Research Involving Human Subjects. The study and its protocols were approved by local ethics committee of the authors’ Hospitals. Informed consents for all clinical details and images publication were taken from all the patients.

### Surgical technique

**Fig. 2:**
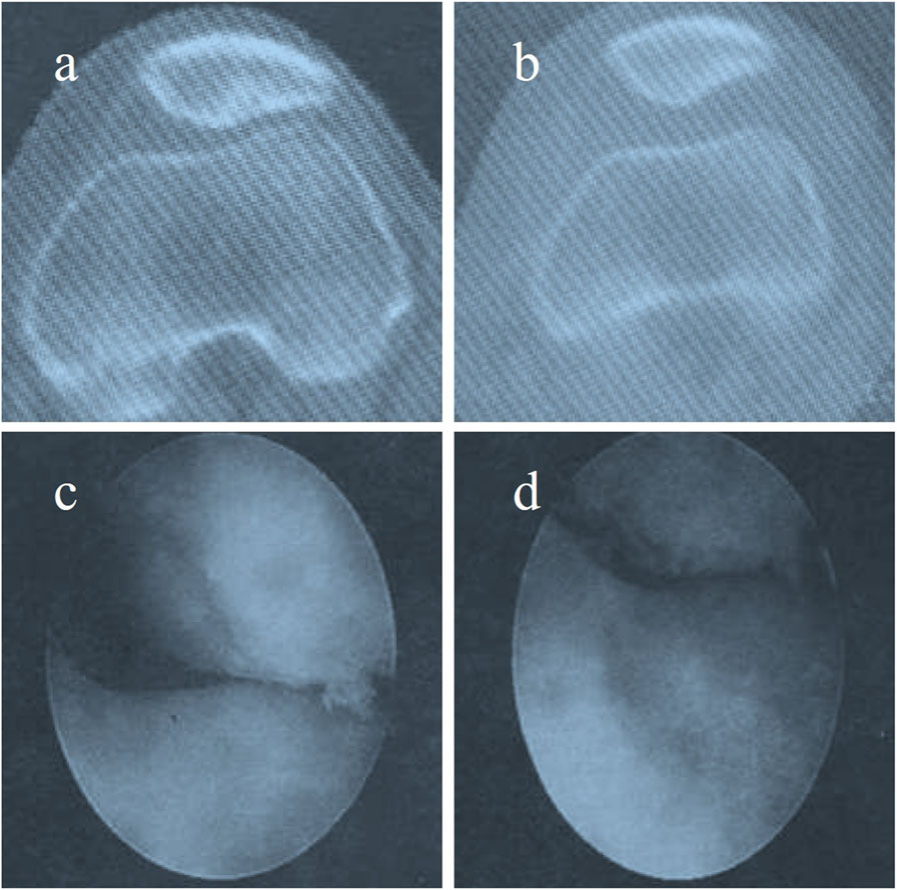
Comparison of preoperative and postoperative radiographic data of CT scan and arthroscopic examination of the ELPS patient. **a** Preoperative CT scan image revealed lateral articular space stenosis. **b** Postoperative CT scan image revealed that the lateral joint space returned to normal. **c** Preoperative arthroscopic examination revealed lateral articular space stenosis. **d** Postoperative arthroscopic examination revealed that the lateral joint space returned to normal

All arthroscopic surgeries were performed by the same group of orthopedic surgeons. The anteromedial and/or anterolateral portals during arthroscopy were applied for dynamic arthroscopic examination of patellofemoral joint. After joint debridement, the shape of the patella and the matching relationship of articular cartilage between the patellofemoral joint were observed. Then, the consistency of the motion trajectory between the patella and trochlear groove during flexion and extension of the knee was observed. The operation process of arthroscopic lateral patelloplasty was consistent with that described by Wu et al. [9]. The lateral margin and facet of the Wiberg types III and IV patella were trimmed and polished via the anterior superolateral portal with a grinding drill to type II or even close to type I. Other knee diseases such as patellar periosteal folds, loose bodies, and hyperplastic bone were removed under arthroscopy. The procedure of arthroscopic LR release was the same as that described by Calpur [10]. The LR release surgery was performed with the anterior superolateral portal that the 2- to 3-cm-long incision was in the lateral and superior edge of the patella and slightly offset from the lateral midline of knee. The LR was bluntly separated, and a working gap was formed on the surface of the LR. Anterior inferolateral portal was inserted into a mirror, and the anterior superolateral portal was used as the instrument inlet. The superficial layer of the LR and the deep layer of the LR, including patellofemoral ligament, patellotibial ligament, and lateral transverse ligament of the patella, were exposed and partially or totally cut off by the planer cutter. Finally, arthroscopic evaluation should be conducted to confirm that the motion trajectory of the patella and the matching relation of patellofemoral articular surface has returned to normal. After the radiofrequency hemostasis, the drainage tube was placed in the released zone and not placed in the joint cavity (Fig. 2).

### Postoperative management

After the surgery, the cotton bandage was evenly pressurized and bandaged. The negative pressure drainage was placed and removed at 24 to 48 h after surgery. After 48 h after surgery, the quadriceps muscle strength training, patella medial push training, and progressive knee joint activity training were started in the brace protection. The knee joint activity was controlled in the range of 0–90° within 2 weeks after surgery and increased to 0–120° at 3–4 weeks after surgery. After 4 weeks, full-scale activity training and muscle strength training were started. After the activity and muscle strength returned to normal, the normal activity was resumed.

### Outcome measurements

The patellofemoral articular cartilage injury was scored by Outerbridge classification [15]. The follow-up was performed to evaluate the changes of subjective symptoms and objective signs. The patellofemoral joint Kujala score, knee function Lysholm score, and visual analogue scale (VAS) were measured before and after surgery [16, 17]. Physical examination included patella impact pain, medial shift of patella, and patella tilt test (ROM) [3, 18–20]. Radiological evaluation of CT scan of knee flexion at 30° included PTA and LPFA [13, 14]. All surgical complications such as incision and joint infection, postoperative hematoma, hemarthrosis, deep vein thrombosis, quadriceps rupture and poor compensation, medial dislocation of the patella, and reflex neurotrophy. were observed discreetly [21].

### Statistical analysis

Continuous variables were expressed as mean ± standard deviation. SPSS 19.0 statistical software (SPSS, Chicago, IL, USA) was used to conduct the paired *t* test. The correlation analysis were investigated by single factor analysis and Logistic regression analysis. *P* ≤ 0.05 was considered statistically difference.

## Results

The articular cartilage injury of the lateral facet of the patella and femoral trochlear groove were scored by the Outerbridge classification (Table 2).

**Table 2:** Cases data and Outerbridge classification

Parameters	Cases
Patients	73
Men	16
Women	57
Years	30-58
Knees	88
Left knees	43
Right knees	45
Outerbridge Classification	
Lateral patella grade II	10
Lateral patella grade III	16
Lateral patella grade IV	62
Lateral trochlear groove grade I	5
Lateral trochlear groove grade II	6
Lateral trochlear groove grade III	37
Lateral trochlear groove grade IV	40

Follow-up ranged from 12 to 36 months with an average of 24 ± 5.8 months. The Lysholm score and Kujala score were significantly increased at the last follow-up compared with before surgery (*P* < 0.01, respectively). The VAS score was significantly lower at the last follow-up than before surgery (*P* < 0.01). The medial pushing distance was significantly increased at the last follow-up compared with pre-surgery (*P* < 0.01). PTA and LPFA was significantly increased at the last follow-up compared with before surgery (*P* < 0.01, respectively) (Table 3).

**Table 3:** Comparison of the observed indexes preoperatively and at the last follow-up

Parameters	Preoperatively	At the last follow-up
VAS	7.12 ± 0.87	1.54 ± 0.79*
Kujala score	59.454 ± 7.59	86.43 ± 9.21*
Lysholm score	54.55 ± 5.74	88.96 ± 7.25*
Patella medial pushing distance	0.66 ± 0.13 cm	1.35 ± 0.26 cm*
TA	2.88 ± 0.41	18.73 ± 0.87*
LPFA	0.98 ± 0.49	8.52 ± 0.67*

*At the last follow-up compared with pre-surgery *P* < 0.01

Years and lateral patella Outerbridge classification have negative correlation with Kujala score, Lysholm score, Patella medial pushing distance, PTA, and LPFA and have positive correlation with VAS at the last follow-up (Tables 4 and 5).

**Table 4:** Results of univariate logistic regression analysis of years for surgical effect assessment

Surgical effect	*β*	*SE*	Wald *χ*^2^	*OR*	*P*
VAS	0.158	0.015	2.159	1.022	0.023
Kujala score	0.017	0.332	0.014	1.017	0.024
Lysholm score	0.010	0.004	6.954	1.010	0.014
Patella medial pushing distance	0.691	0.267	0.142	1.147	0.011
PTA	0.137	0.212	0.408	0.657	0.022
LPFA	0.428	0.229	0.197	0.501	0.010

**Table 5:** Results of univariate logistic regression analysis of lateral patella Outerbridge classification for surgical effect assessment

Surgical effect	*β*	*SE*	Wald *χ*^2^	*OR*	*P*
VAS	0.007	0.004	3.322	1.007	0.028
Kujala score	1.214	0.378	11.748	3.032	0.004
Lysholm score	1.082	0.345	8.563	2.213	0.025
Patella medial pushing distance	1.313	0.423	7.478	1.986	0.035
PTA	1.215	0.498	9.324	2.554	0.020
LPFA	1.657	0.524	9.128	4.576	0.022

Surgical complications were not reported. Manipulation under anesthesia for knee contracture due to postoperative adhesion was required in only 3 patients.

## Discussion

LR release surgery for hook-shaped patella can reduce the pressure on the lateral facet of the patellofemoral joint to a certain extent, but it cannot correct lateral patella morphology and eliminate lateral impact of the patellofemoral joint. Therefore, we used the extra-articular LR release surgery combined with intra-articular lateral patelloplasty for the intractable ELPS with hook-shaped patella, that is, grinding the outer edge and articular facet of the patella to make it a type I or type II patella. In our study, the Kujala score, Lysholm score, patella medial pushing distance, PTA, and LPFA were significantly increased and VAS was significantly decreased at the last follow-up compared with before surgery, which manifests that arthroscopic LR release combined with lateral patelloplasty could effectively restore the mechanical structure and motion trajectory of the patella, improve patellar tracking and patella mobility, ultimately improve patellofemoral joint function, and effectively relieve anterior knee pain. Besides, years and lateral patella Outerbridge classification have negative correlation with Kujala score, Lysholm score, patella medial pushing distance, PTA, and LPFA and have positive correlation with VAS at the last follow-up. These results suggest that younger patients or patients with less cartilage damage are more likely to achieve good surgical effect.

Anterior knee pain is the most common complaint in ELPS [13]. ELPS was first described by Ficat et al. in 1975 [22]. The patients were mainly suffering from anteriolateral pain of knee joint, which was obvious as patella weight raise such as going down and up stair and squatting. The tightened LR produces high pressure on the lateral surface of the patellofemoral joint, which can soften and even exfoliate cartilage. The elevated pressure stimulates the nerves of the subchondral bone to cause pain. Arthroscopic LR release combined with lateral patelloplasty can relax the contracture of LR, repair the deformation of the lateral patellofemoral joint surface, and reduce the lateral articular surface pressure. Therefore, in our study, VAS was significantly lower at the last follow-up than before surgery, suggesting that anterior knee pain was significantly relieved. Zhu et al. [23] reported that LR release can reduce postoperative knee pain without increasing complications in the treatment of unilateral degenerative knee arthritis after total knee arthroplasty without resurfacing of the patella.

Patella can be moved to the medial side at least 15 mm under normal circumstances, but the medial mobility in ELPS is obviously reduced and less than 1/4 of the patella length [24]. Arthroscopic LR release combined with lateral patelloplasty reduces the LR tension and balances the lateral and medial articular surface tension of patellofemoral joint, which results in improved patella activity. So, in our study, patella medial pushing distance was significantly higher at the last follow-up than before surgery.

The patella in ELPS is displaced outward and tilted. Imaging examination shows that PTA and LPFA are obviously reduced. The medial and lateral articular surface tension of patellofemoral joint is significantly asymmetrical. Moreover, some patients are accompanied by patella subluxation [3]. Long-term oblique compression of lateral patella will destroy articular cartilage [13]. The LR release can correct the abnormally inclined patella, so lateral patella articular surface can be protected from damage [25, 26]. Arthroscopic LR release can reduce the tension of LR and restore patellar stress balance inside and outside. Lateral patelloplasty repairs abnormal patella morphology and restore patellofemoral joint alignment. So, the combination of the two procedures will try to restore the patella trajectory to normal. Therefore, in our study, PTA and LPFA were significantly increased at the last follow-up compared with that before surgery. In addition, we also found that years and lateral patella Outerbridge classification have negative correlation with Kujala score, Lysholm score, patella medial pushing distance, PTA, and LPFA and have positive correlation with VAS at the last follow-up, which suggests that younger patients or patients with less cartilage damage are more likely to achieve good surgical effect. So, early treatment can effectively prevent further damage of the articular cartilage. Once the cartilage is seriously damaged, the treatment effect is not very effective. Therefore, we advocate early surgery of LR release for patients with ELPS.

Verdonk et al. [25] compared the results of knee CT examination before and after LR release surgery and found that the procedure can effectively improve the morphology of the patient’s patellofemoral joint. Arthroscopic closing LR release for the treatment of ELPS can effectively improve the function and relieve symptoms of patellofemoral joint with the advantages of small trauma, rapid recovery and less complications [27]. In our study, similar results were found that Kujala score and Lysholm score were significantly increased at the last follow-up compared with that before surgery, indicating significant improvement in knee function. In other study, the arthroscopic LR release in the treatment of a symptomatic type III bipartite patella had significantly improved Kujala and VAS scores with excellent symptom relief [28].

Arthroscopic LR release is often performed from the inside to the outside of the patellofemoral joint. The synovial membrane, joint capsule, and LR were partially or totally cut off, which easily caused complications of intra-articular hemorrhage and joint adhesion, which affected curative effect [10]. Therefore, we performed LR release from the surface of LR to preserve synovium and capsule of joint, so as to reduce the risk of joint hematoma and adhesion [27]. In our study, manipulation under anesthesia for knee contracture due to postoperative adhesion was required in only 3 patients, and joint hematoma has not been reported.

The following points are important intraoperatively. Intraoperative attention should be paid to dynamic and visual observation of the patella motion trajectory, patellofemoral joint surface contact pressure, and patella tilt. Besides, the osteophyte around patellofemoral joint affecting patella movement trajectory should be removed. The bare joint surface should be ground fresh being beneficial to the formation of fibrocartilage [29]. The shallow and deep layer of LR should be carefully identified to prevent underrelease and overrelease. Hemostasis of arthroscopic electrocoagulation should be complete and sufficient to prevent hematomas and adhesions. Finally, the LR is partially or completely released according to the severity of the patella tilt. The hook-shaped patella should be reshaped to form a V-shaped patella according to the shape of the femoral trochlear groove. We have realized that this surgery has the following advantages (Table 6).

**Table 6:** the advantages of arthroscopic LR release combined with lateral patelloplasty

The advantages	
①Arthroscopic LR release has small trauma, conducive to the postoperative recovery and early functional exercise. Intraoperative dynamic observation of the patellofemoral joint surface can guide the degree of the release and observe the released effect. ②The reservation of the synovial membrane and joint capsule during arthroscopic LR release can reduce postoperative adhesions and intra-articular hemorrhage, and prevent excessive release. ③Lateral patelloplasty could correct lateral patella morphology and improve patellofemoral joint matching and motion trajectory of patella, and reduce lateral impact between the lateral articular surface of the patellofemoral joint to reduce secondary cartilage damage. ④Direct vision during surgery does prevent injury to the lateral femoral muscle tissue, and can selectively release LR shallow and deep layers partially or totally, which could prevent over-release. ⑤Surgeons could dynamically and intuitively observe the motion trajectory of the patella, contact pressure of the patellofemoral joint and the degree of degeneration of the affected articular cartilage so as to facilitate accurate judgment of the disease and timely observation of the surgical effect.	

The strength of this study is the first use of arthroscopic LR release combined with lateral patelloplasty in the treatment of ELPS, which can simultaneously correct abnormalities of line of force and skeleton. But, this study has some limitations which should be reported. The sample size was small. Due to relatively short follow-up time, the long-term outcomes of this surgery were not obtained. No control group was set. A follow-up study comparing the simple arthroscopic LR release or arthroscopic arthroplasty with arthroscopic lateral LR release combined with lateral patella arthroplasty for the treatment is urgently needed. Besides, this study has a large age span and different grade of cartilage damage, which may bias the results and require further standardization. Due to the limitation of experimental conditions, this study lacks the most powerful biomechanical research. So the results need further research and argumentation.

## Conclusion

Full arthroscopic LR release combined with lateral patelloplasty in the treatment of ELPS is an effective minimally invasive method, which can effectively correct anomalies of force line and structure, relieve pain, and restore knee joint motor function with less complications.

## Data Availability

The datasets used and/or analyzed during the current study are available from the corresponding author on reasonable request.

## Abbreviations

ELPS: Excessive lateral pressure syndrome
LR: Lateral retinaculum
PTA: Patellar tilt angle
LPFA: Lateral patellofemoral angle
VAS: Visual analogue scale
